# Research on Fano Resonance Sensing Characteristics Based on Racetrack Resonant Cavity

**DOI:** 10.3390/mi12111359

**Published:** 2021-11-03

**Authors:** Yaxin Yu, Jiangong Cui, Guochang Liu, Rongyu Zhao, Min Zhu, Guojun Zhang, Wendong Zhang

**Affiliations:** State Key Laboratory of Dynamic Testing Technology, North University of China, Taiyuan 030051, China; s1906103@st.nuc.edu.cn (Y.Y.); jgcui@nuc.edu.cn (J.C.); b200611@st.nuc.edu.cn (G.L.); s2006241@st.nuc.edu.cn (R.Z.); s2006170@st.nuc.edu.cn (M.Z.); wdzhang@nuc.edu.cn (W.Z.)

**Keywords:** metal-insulator-metal, racetrack resonant cavity, Fano resonance, refractive index sensing, temperature sensing

## Abstract

To reduce the loss of the metal–insulator–metal waveguide structure in the near-infrared region, a plasmonic nanosensor structure based on a racetrack resonant cavity is proposed herein. Through finite element simulation, the transmission spectra of the sensor under different size parameters were analyzed, and its influence on the sensing characteristics of the system was examined. The analysis results show that the structure can excite the double Fano resonance, which has a distinctive dependence on the size parameters of the sensor. The position and line shape of the resonance peak can be adjusted by changing the key parameters. In addition, the sensor has a higher sensitivity, which can reach 1503.7 nm/RIU when being used in refractive index sensing; the figure of merit is 26.8, and it can reach 0.75 nm/°C when it is used in temperature sensing. This structure can be used in optical integrated circuits, especially high-sensitivity nanosensors.

## 1. Introduction

Surface plasmon polaritions (SPPs) are evanescent waves propagating along the surface of a metal, formed by the coupling of free electrons and photons on the metal’s surface [1,2,3,4,5]. They propagate along the metal–medium surface and decay exponentially on both sides vertical to the interface. SPPs can overcome the limitations of the traditional optical diffraction limit and manipulate light waves in the sub-wavelength range; thus, they are widely applied in highly integrated nano-optoelectronic devices, such as nanosensors [6,7], filters [8,9,10], and waveguide modulators [11].

In recent years, the plasmon-induced transparency (PIT) [12], plasmon-induced absorption (PIA) [13], and Fano resonance [14] in surface plasmon nano-integrated sensor devices have attracted considerable research attention in the field of nano-integration. Among them, Fano resonance [15] is a phenomenon in which zero absorption occurs in the absorption spectrum, owing to the coupling interference effect between the continuous and discrete state energy levels. This interference effect exhibits asymmetric resonance that is different from the traditional Lorentz line spectrum. Because this asymmetrical Fano resonance spectrum has sharp lines, low radiation loss, and the resonance frequency is highly correlated with the refractive index and structural parameters of the filling material, it can be applied to the field of nanosensing with a higher figure of merit (FOM) and sensitivity (S) [16,17]. Therefore, Fano resonance is widely used in sensors [18], optical switches [19], slow light devices [20], and other fields.

In recent years, nanosensor structures of different shapes based on Fano resonance have been proposed by researchers, such as cup cavity [21], splitting ring cavity [22], notched cavity [23], and multiple ring cavity [24]. These structures can easily integrate and contact the sensing medium. Improving these structures to enhance sensitivity has increasingly become a major focus for researchers. For example, Liu et al. designed a sensor structure with a short rod and a cup-shaped resonant cavity coupling [21], which serves as a refractive index sensor with a sensitivity of 600 nm/RIU. Zhang et al. proposed a refractive index sensor structure coupled with a tooth-shaped cavity [22], whose sensitivity reached 1200 nm/RIU, whereas Liu et al. analyzed a sensor structure coupled with a hexagonal resonant cavity [25] as a refractive index sensor that achieved a sensitivity of 937 nm/RIU. Chen et al. proposed a sensing system coupled with a rectangular resonant cavity [26] with a temperature-sensing sensitivity of 0.225 nm/°C. Chen et al. discussed temperature-sensing characteristics based on the ring resonant cavity [27], the sensitivity of which reached 0.31 nm/°C.

A metal–insulator–metal (MIM) waveguide coupled racetrack resonant cavity (RTRC) nanosensor with a baffle is proposed to reduce the loss of the MIM waveguide structure in the near-infrared region. To enhance the sensitivity of the sensor, defects are introduced into the structure to destroy its symmetry. The structure proposed in this study is simple, sensitive, and easy to manufacture. It is not necessary to change both the horizontal and vertical sizes of the ring cavity in the racetrack resonant cavity. The size of the raceway can be adjusted to meet the requirements of some special-sized devices. The influence of the structural parameters and the refractive index of the surrounding medium on the transmission characteristics is studied using the finite element method. Finally, the structural parameters are optimized, and a high sensitivity and figure of merit (FOM) are obtained. The structure potentially has great applications in optical communications and high-density integrated optics, especially in nano-optical sensing.

## 2. Model Building and Theory

### 2.1. Model Building

**Figure 1:**
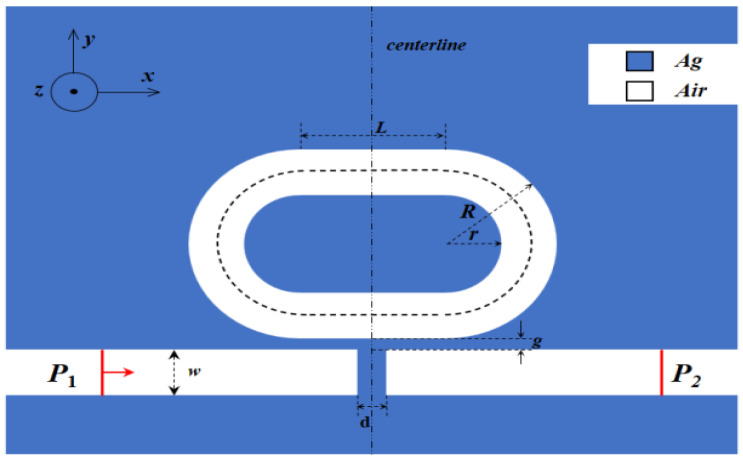
Schematic diagram of two-dimensional structure of plasmonic nanosensor.

The proposed RTRC structure and its geometric parameters are illustrated in Figure 1. The structure consists of a baffled MIM waveguide and a racetrack resonator. [28], It can be fabricated by electron beam lithography and a stripping process during the experimental preparation, which comprises the following steps: (1) a metal layer is plated on the substrate—non-absorbent materials such as silicon dioxide can be selected as the substrate; (2) the waveguide structure is etched onto the metal layer using electron beam lithography and a stripping process [29,30]. The blue part in Figure 1 represents metallic Ag, and the white part represents the air medium. The relative permittivity of air, \varepsilon_d = 1, and the relative permittivity of Ag are related to the frequency of the incident light. The dielectric constants calculated by the Debye-Drude model are in good agreement with experimental data in the red and near-infrared portions of visible light (600 nm–2000 nm), According to the Debye-Drude dispersion model [31]:

(1) $$\varepsilon \left(\omega \right)=\varepsilon_{\infty }+\frac{\varepsilon_{s}-\varepsilon_{\infty }}{1+i\omega \tau }+\frac{\sigma }{i\omega \varepsilon_{0}}$$

where $\varepsilon_{0}$ represents the dielectric constant in a vacuum; ω represents the angular frequency of the incident light waves in a vacuum; $\varepsilon_{\infty }$ represents an infinite dielectric constant, where $\varepsilon_{\infty }=3.8344$; $\varepsilon_{s}$ represents the static dielectric constant, where $\varepsilon_{s}=-9530.5$; τ represents the damping coefficient,$\;\tau =7.35\times 10^{-15\;}s$; σ represents Ag conductivity, $\;\sigma =1.1486\times 10^{7}\;S/m$.

A two-dimensional geometric model was established using the finite element (FEM) software COMSOL Multiphysics 5.5, and the width of the MIM waveguide and racetrack resonant cavity was set to *w* = 50 nm to ensure that only the basic transverse magnetic mode (TM0) was supported [32]. The length of the racetrack was set to *L* = 120 nm, and the outer diameter of the resonant cavity was *R* = 140 nm. The relationship between the outer diameter *R* and the inner diameter r was *R = r* + 50 nm, the baffle width was *d* = 20 nm, and the coupling distance was *g* = 10 nm. The boundary conditions around the structure are set as a perfect matching layer (PML) with a thickness of 100 nm. The incident light enters from the left port of the model and is output from the right port. The grid adopts ultra-fine grid, the parameterized scanning interval is 1 nm, and the electromagnetic wave frequency domain analysis module is used to numerically analyze the transmission spectrum and mode field distribution of the sensor system.

### 2.2. Fano Resonance Principle Analysis

**Figure 2:**
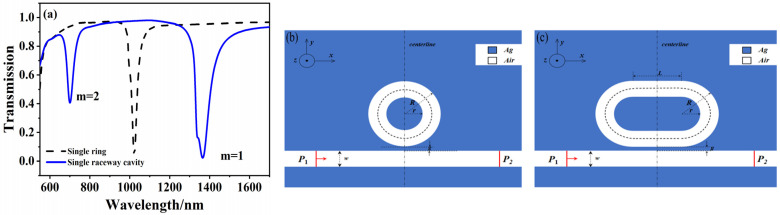
Comparison of transmittance spectrum between a circular ring and a RTRC side-coupled to a bus waveguide (**a**) Transmission pattern. (**b**) Schematic diagram of the structure of the ring. (**c**) schematic diagram of the structure of the raceway cavity.

Firstly, the effect of the introduced racetrack structure is analyzed by simulation. The transmission spectrum of the coupling ring on the side of the MIM waveguide without the baffle, and that of the coupling ring and the coupled RTRC on the MIM waveguide side are shown in Figure 2a. The analyzed structure diagram is shown in Figure 2b,c. Compared with the circular ring, the RTRC transmission spectrum has more transmittance drop valleys, and new resonance modes appear at λ = 700 nm and λ = 1365 nm, which are expressed as m = 2 and m = 1, respectively.

Based on the coupled mode theory (CMT) [25], the mechanism of double Fano resonance is analyzed. The resonant amplitudes of the two resonant modes in the racetrack resonant cavity are represented by $f_{1}$ and $f_{2}$, and normalized to the energy in the resonant cavity; $S_{1+}$ and $S_{2+}$ respectively represent the mode field amplitudes of the incident light at the incident port and the output port, $S_{1-}$ and $S_{2-}$ respectively represent the mode field amplitude of the light emitted from the incident port and the output port. With the change of time, the amplitude of the transmitted wave in the resonant cavity is [26]:

(2) $$j\omega f_{1}=\left(j\omega_{1}-\frac{1}{\tau_{o1}}-\frac{1}{\tau_{e1}}\right)f_{1}+k_{1}\left(S_{1+}+S_{2+}\right)$$

(3) $$j\omega f_{2}=\left(j\omega_{2}-\frac{1}{\tau_{o2}}-\frac{1}{\tau_{e2}}\right)f_{2}+k_{2}\left(S_{1+}+S_{2+}\right)$$

wherein, ω is the angular frequency of the incident light, $\omega_{1,2}$ corresponds to the resonant frequencies of the two resonant modes, $\frac{1}{\tau_{oi}}$(i=1,2) represents the natural frequencies of the two modes of the resonant cavity, and $\frac{1}{\tau_{ei}}\left(i=1,2\right)$ corresponds to the amplitude attenuation of the two modes of the resonant cavity respectively coupled to the waveguide, and $k_{i}\left(i=1,2\right)$ correspond to the input coupling coefficients of SPPs wave propagating forward and backward in the waveguide respectively. According to the law of conservation of energy [27], the output wave can be expressed as:

(4) $$\left(\begin{array}{c} S_{1-}\\ S_{2-} \end{array}\right)=C\left(\begin{array}{c} S_{1+}\\ S_{2+} \end{array}\right)+k\left(\begin{array}{c} f_{1}\\ f_{2} \end{array}\right)$$

wherein, C represents the scattering matrix directly coupled between the incident wave and the outgoing wave at the baffle in the waveguide structure. The expression is:

(5) $$C=\left(\begin{array}{cc} r & jt\\ jt & r \end{array}\right)$$

wherein, t and r respectively correspond to the coefficients of the transmission amplitude and reflection amplitude, and r+t=1; k is the coupling matrix between the output wave and the output port, and the specific expression is as follows:

(6) $$k=\left(\begin{array}{cc} -k_{1}^{*} & k_{2}^{*}\\ -k_{1}^{*} & -k_{2}^{*} \end{array}\right)$$

From the time-reversal composability and the law of conservation of energy, we can get: $k_{1}=\sqrt{1/\tau_{e1}}e^{j\theta_{1}}$, $k_{2}=\sqrt{1/\tau_{e2}}e^{j\theta_{2}}$. Wherein, $\theta_{1}$ and $\theta_{2}$ respectively correspond to the phase coupling coefficients of the two coupling modes. The light transmission passes through the waveguide structure system, and the transmission spectrum amplitude is defined as $t\left(\omega \right)=S_{2-}/S_{1+}$.The calculation expression of the transmittance is:

(7) $$T\left(\omega \right)={\left|t\left(\omega \right)\right|}^{2}={\left|jt-\frac{\frac{1}{\tau_{e1}}}{j\left(\omega -\omega_{1}\right)+\frac{1}{\tau_{o1}}+\frac{1}{\tau_{e1}}}-\frac{\frac{1}{\tau_{e2}}}{j\left(\omega -\omega_{2}\right)+\frac{1}{\tau_{o2}}+\frac{1}{\tau_{e2}}}\right|}^{2}$$

From Equation (6), it can be concluded that in the absence of a baffle r=0, t=1, and $\frac{1}{\tau_{o1}}$,$\frac{1}{\tau_{o2}}$, $\frac{1}{\tau_{e1}}$ and $\frac{1}{\tau_{e2}}$ are all constants, which are far less than $\left|j\left(\omega -\omega_{1.2}\right)\right|\left(\omega \neq \omega_{1,2}\right)$. At this time, the transmittance is larger, and when the incident light frequency ω increases, its value is close to or equal to $\omega_{1.2}$, the transmittance suddenly drops sharply, forming two narrower discrete spectra. Therefore, compared with the regular ring structure, this study attributed the emergence of the new resonance mode to the introduced racetrack structure.

**Figure 3:**
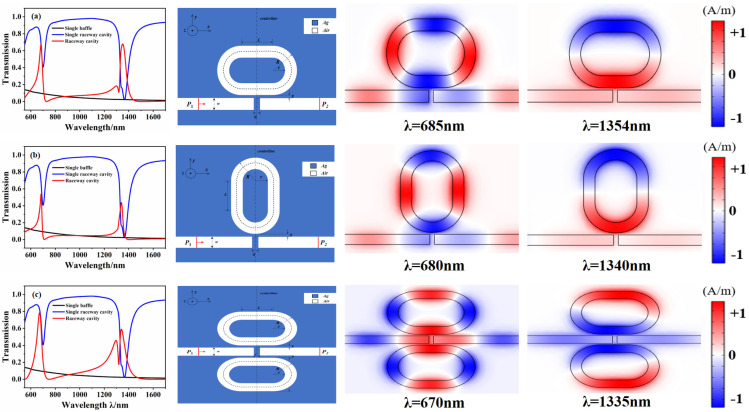
Transmission spectrum, structure diagram, and the Hz field distribution diagram of resonance point of RTRC under different placement methods. (**a**) Horizontal placement. (**b**) Vertical placement. (**c**) Symmetrical placement.

As shown in Figure 3, to verify the effect of the introduced baffle, a silver baffle was inserted into the MIM waveguide, where the width of the baffle was 20 nm and the other parameters were the same as those in Figure 2b. The entire sensor system was formed using the RTRC. The transmission spectra under different placement conditions are presented in Figure 3.

The transmission spectrum of the sensing system when placed horizontally is shown in Figure 3a. When the sensor system (red line) is at λ = 685 nm and λ = 1354 nm, a sharp asymmetric line is observed, which rapidly drops from the peak to the trough. This is a typical Fano resonance phenomenon. Here, the sensor system is disassembled into two structures, and the formation principle of Fano resonance is analyzed. The transmission spectrum for the structure of the coupled RTRC on the side of the MIM waveguide without baffle is represented by the blue solid line. The analysis showed a narrower transmittance inclination angle, which appeared as a discrete state. Conversely, the transmission spectrum for the baffled MIM waveguide with an RTRC-free structure is represented by the black solid line. A broad and continuous transmission spectrum is shown, with a negative slope and a low transmittance. The Fano phenomenon can therefore be explained by the interaction between a wide, continuous state and a narrow, discrete state. The peaks of Fano resonance are λ = 685 nm and λ = 1354 nm, and the corresponding magnetic field distribution is also shown in the figure. From the magnetic field distribution, it is concluded that this is a symmetrical mode with m = 2 and m = 1.

The transmission spectrum of the sensor system when placed vertically is shown in Figure 3b. When the sensor system (red line) is at λ = 680 nm and λ = 1340 nm, there is also a sharp asymmetrical line that rapidly drops from the peak to the trough. Again, this is typical of the Fano resonance phenomenon, but the resonant peaks have transmittances of 0.54 and 0.42, which are lower than the transmittances of 0.69 and 0.71 in the horizontal placement mode. From the steady-state magnetic field distribution diagram, a symmetrical mode with m = 2 and m = 1 can be seen. The transmission spectrum of the sensing system when placed symmetrically is shown in Figure 3c. It is clear that the Fano resonance occurs at λ = 670 nm and λ = 1335 nm. The transmittances of the two resonance peaks are 0.78 and 0.59, which are better than those of the vertical placement method, but the values are generally lower than those of the horizontal placement method. The magnetic field distribution diagram shows that the two resonance peaks, λ = 685 nm and λ = 1340 nm, correspond to the symmetric mode with m = 2 and m = 1.

The analysis shows that the introduction of the track structure can achieve a double Fano resonance. Different placement methods can affect the Fano resonant peak, whereas the position of the resonant peak is less affected. Simultaneously, the Fano resonant peak is sharper in the horizontal placement mode. In the subsequent discussion, the processing technology was mainly based on the structure in the horizontal placement mode.

## 3. Structural Analysis

The different structural parameters of the racetrack resonant cavity affect the position of the resonant peak and its peak value. Therefore, the transmission characteristics of the sensing system are related to the geometric parameters of the structure, meaning the transmission spectra under different structural parameters must be analyzed. The performance of the sensing system generally has two important evaluation indicators, usually expressed by S and FOM [23]:

(8) $$S=\frac{\Delta \lambda }{\Delta n}\left(nm/RIU\right)$$

(9) $$FOM=\frac{S}{FWHM}$$

where Δn is the change in refractive index, Δλ is the change in the resonance wavelength, and FWHM is the full width at half maximum.

**Figure 4:**
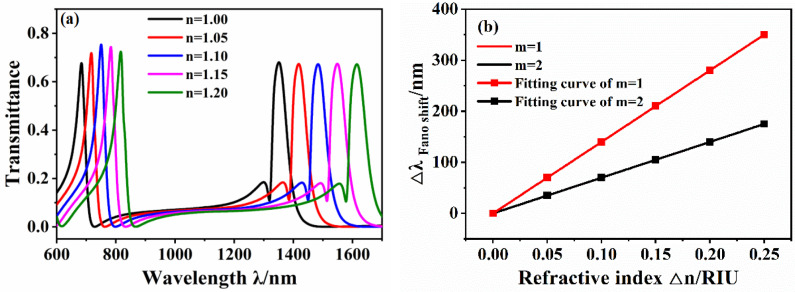
(**a**) Transmission spectrum of waveguide structure under different refractive index n. (**b**) Sensitivity fitting curve.

The refractive index-sensing performance of the structure was analyzed by calculating the system transmission spectra under different refractive indices n. The structural parameters of the waveguide were the same as those in Figure 1, and the refractive index was increased from 1 to 1.20 in intervals of 0.05. Figure 4a shows the transmission spectra of the waveguide structure under different media refractive indices. As shown in the figure, the Fano resonances in both modes of the transmission spectra show a redshift as the refractive index *n* increases.

The sensitivity of using the linear fitting of the two symmetrical modes is shown in Figure 4b. The sensitivity of symmetrical mode m = 1 is 1401.3 nm/RIU, FWHM = 53.0 nm, with FOM = 26.4, and the sensitivity of symmetrical mode m = 2 is 701.2 nm/RIU, FWHM = 15.14 nm, with FOM = 46.31. The analysis results show that the structure has a higher sensitivity as a refractive index sensor and can therefore be used in the field of refractive index sensing. The comparison shows that the sensitivity and FOM in the m = 1 mode are higher, and the symmetric mode m = 1 is discussed later.

**Figure 5:**
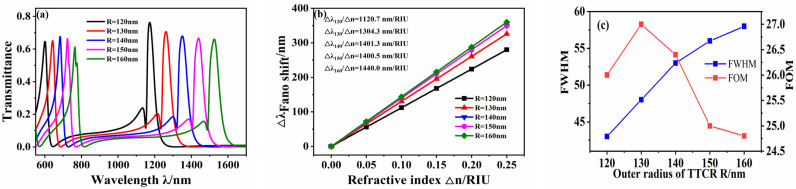
(**a**) Transmission spectrum of waveguide structure under different *R*. (**b**) Sensitivity fitting curve. (**c**) Fitting curve of FWHM and FOM values.

Other structural parameters of the fixed waveguide structure remained unchanged, such that *L* = 120 nm, *g* = 10 nm, and *d* = 20 nm, and the outer diameter *R* of the RTRC was increased from 120 nm to 160 nm in intervals of 10 nm to investigate the influence of a single variable—the RTRC outer diameter—on the transmission spectrum. The transmission spectra of the structure under different outer diameters were obtained from the simulation, as shown in Figure 5a. With an increase in *R*, the Fano resonance exhibits a significant redshift. The m = 1 mode corresponds to a weakening Fano resonance peak. This is attributed to the change in *R* affecting the change in the narrowband mode, where this corresponding change in the narrowband mode leads to changes in the position and transmittance of the Fano resonance [33]. The change in the Fano resonance peak with the refractive index Δn under the symmetry mode m = 1 is shown in Figure 5b. When *R* = 160 nm, the maximum sensitivity can reach 1440 nm/RIU, and the FOM is 27.

**Figure 6:**
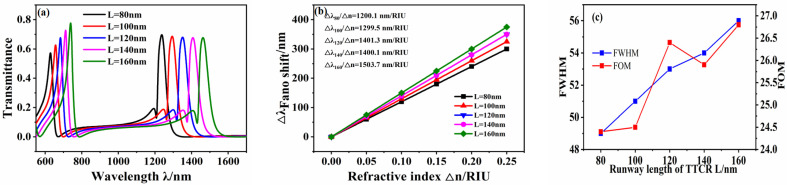
(**a**) Transmission spectrum of waveguide structure under different *L*. (**b**) Sensitivity fitting curve. (**c**) Fitting curve of FWHM and FOM values.

In addition, the introduced racetrack structure affects the transmission performance of the sensing system, and therefore, its size parameters are analyzed. The length *L* of the racetrack was increased from 80 nm to 160 nm in intervals of 20 nm, and other parameters were fixed at *R* = 140 nm, *g* = 10 nm, and *d* = 20 nm. The transmission spectra of the sensor system for different racetrack lengths are shown in Figure 6a. With an increase in *L*, the Fano resonance produces a significant redshift, and the intensity of the Fano resonance peak on the right gradually decreases. This is because the position of the Fano resonance is determined by the narrowband discrete state, and the increase in *L* triggers a decrease in the resonance wavelength of the narrowband spectrum, so that the Fano resonance produces a redshift. The wavelength drift of the Fano resonance peak with the refractive index Δn under symmetry mode m = 1 is shown in Figure 6b. After fitting, the maximum sensitivity of the system reaches 1503.7 nm/RIU when *L*= 160 nm, and the FOM is 26.8.

**Figure 7:**
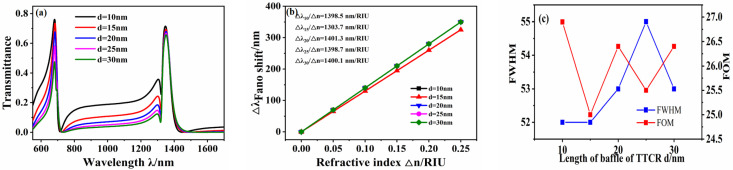
(**a**) Transmission spectrum of waveguide structure under different *d*. (**b**) Sensitivity fitting curve. (**c**) Fitting curve of FWHM and FOM values.

The width of the introduced baffle also affects the transmission spectrum of the sensor system. The other parameters were fixed at *R* = 140 nm, *r* = 90 nm, *L* = 120 nm, and *g* = 10 nm, and the baffle width d was increased from 10 nm to 30 nm in intervals of 5 nm. The transmission spectra under different baffle widths were simulated, as shown in Figure 7a. As the baffle width d increases, there is no significant change in the position of the Fano resonance peak. The fitting curve of the displacement of the Fano peak with the refractive index Δn under the symmetry mode m = 1 is shown in Figure 7b, which illustrates that the sensitivity of the sensor system only slightly changes with the increase in *d*. The FWHM first increases and then decreases, and the FOM value does not change significantly, indicating that the baffle width has little effect on the sensitivity.

By analyzing the transmission spectrum lines under different radii *R*, racetrack lengths *L*, and baffle widths, the influence of these parameters on the sensing system could be investigated. The results show that the transmission spectrum line is most significantly influenced by the radius *R* and the racetrack length *L*. The width did not affect the transmission spectra. When *R* = 160 nm, the sensitivity of the sensing system reaches a maximum of 1503.7 nm/RIU. Compared with previous studies, as shown in Table 1, the designed sensor has the advantage of high sensitivity when applied to refractive index sensing.

## 4. Sensing Application

The waveguide and the RTRC structure are filled with high-refractive-index temperature coefficient materials, and the application of the proposed structure in the field of temperature sensing is explored. Ethanol is the selected filling material owing to its high-refractive-index temperature coefficient (3.94 × 10^−4^), which far exceeds that of Ag ($9.30\times 10^{-6}$) and the substrate material SiO_2_ ($8.60\times 10^{-6}$), and there is an unambiguous linear relationship between the refractive index of ethanol and the temperature:

(10) $$n=1.36048-3.94\times 10^{-4}\left(T-T_{0}\right)$$

where $T_{0}$ is the room temperature taken to be 20 °C, and T is the surrounding temperature to be measured [37]. The variation in the outside temperature mainly affects ethanol, hence the waveguide and RTRC are filled with ethanol. The sensitivity of temperature sensing can be defined as $S_{T}=\Delta \lambda /\Delta T$, where Δλ is the shift in wavelength and ΔT is the variation in temperature. The geometrical parameters of the structure are consistent with those shown in Figure 1.

**Figure 8:**
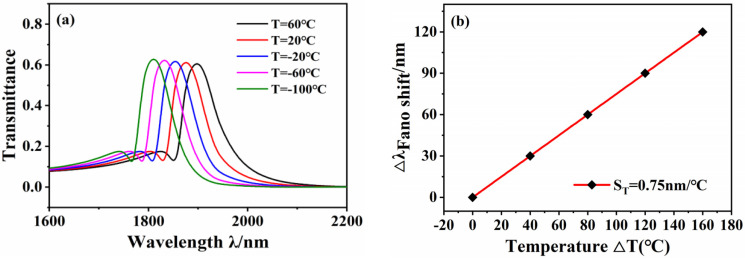
(**a**) Transmission spectrum at different temperatures. (**b**) Temperature sensitivity fitting curve.

The transmission spectra of the structures at different temperatures are shown in Figure 8a. As the temperature increases, the Fano resonance produces a blueshift with an increase in the refractive index. The line type of the Fano resonance remains unchanged, and the height of the resonance peak does not change significantly. According to Equation (9), the temperature is inversely proportional to the refractive index. The Fano resonance is redshifted as the refractive index increases, meaning the Fano resonance is blueshifted as the temperature increases. In addition, the sensitivity fitting curve of the temperature sensor is shown in Figure 8b, and the temperature sensitivity is 0.75 nm/°C. Comparisons with previous studies, as shown in Table 2, show that the designed structure has the advantage of high sensitivity when applied to temperature sensing.

## 5. Conclusions

In summary, this study proposed a sensing system coupled with a baffled MIM waveguide and a racetrack resonant cavity, which was analyzed using the finite element method. The results show that the waveguide structure can achieve double Fano resonance, and different placement methods affect the resonant peak of the sensing system. The design structural parameters, such as radius and racetrack length, simultaneously affect the position and peak values of the Fano resonance peak. In addition, the performance of the sensor is optimized by controlling the geometric parameters. The results show that when the sensing system is used in the field of refractive index sensing, the sensitivity can reach 1503.7 nm/RIU, and the FOM is 26.8; when applied to temperature sensing, the sensitivity can reach 0.75 nm/°C, which is 10 times higher than that of the same type of fibre-optic sensor. It is 10 times more sensitive than the same type of fibre optic sensor. This study introduces a runway structure and a stub structure, which, compared to the regular ring structure, does not require any change in the radius of the resonant cavity, and only requires a dimensional adjustment of the introduced runway to meet the requirements of some special size devices, compared to other ring structures, such as the introduction of defects, stubs, etc. The proposed structure is more compact to prepare and the preparation process is less difficult, thus offering potential for application in the field of optical integration.

## Figures and Tables

**Table 1:** The RI sensitivity reported in references.

References	Structure	Sensitivity (nm/RIU)	FOM (RIU-1)	Resonant Wave Length (nm)
[21]	Cup cavity	600	120	600–1200
[23]	Notched cavity	1071	12.5	500–1700
[22]	Splitting ring cavity	1200	12.2	800–1450
[24]	Multiple ring cavity	1217	24.34	500–1200
[34]	Rectangular cavity	985	25	600–1000
[35]	Multi-sided ring cavity	1949	29.52	100–1600
[36]	Nano ring cavity	2080	29.92	1800–2600
This study	TCRC	1503.7	26.8	600–1500

**Table 2:** The temperature sensitivity reported in references.

References	Structure	Temperature Sensitivity (nm/°C)	Resonant Wavelength (nm)	Sensitive Material
[38]	Fiber-optical sensor	0.073	1500–1600	Water
[39]	Fiber-optical sensor	0.059	1500–1600	Sodium chloride
[40]	Ring cavity	0.62	2600–4400	Ethanol
[41]	Circular cavity	0.664	800–2000	Ethanol
[42]	Array cavity	0.84	800–4000	Ethanol
This study	TCRC	0.75	1600–2200	Ethanol

## Data Availability

The available data has been stated in the article.
